# Proprietary alpha-amylase inhibitor formulation from white kidney bean (*Phaseolus vulgaris* L.) promotes weight and fat loss: a 12-week, double-blind, placebo-controlled, randomized trial

**DOI:** 10.1038/s41598-024-63443-8

**Published:** 2024-06-03

**Authors:** Ralf Jäger, Sidney Abou Sawan, Martin Purpura, Barbara Grube, Yvette Röske, Patricia De Costa, Pee-Win Chong

**Affiliations:** 1 Increnovo LLC, Whitefish Bay, WI USA; 2 Iovate Health Sciences International, Oakville, ON Canada; 3 Practice for General Medicine, Berlin, Germany; 4 Analyze and Realize GmbH, Berlin, Germany; 5 InQpharm Consumer Health Ltd., Abertillery, UK

**Keywords:** Weight management, BMI, Body shaping, Fat mass, Obesity, Physiology, Metabolism

## Abstract

White kidney bean (*Phaseolus vulgaris* L.) extracts can aid weight management by reducing calorie intake from complex carbohydrates through alpha-amylase inhibition. We examined the impact of a proprietary aqueous extract from whole dried white kidney beans standardized by its alpha-amylase inhibitor activity (Phase 2 white kidney bean extract (WKBE)) on weight management in subjects with overweight and moderate obesity. In a randomized, double-blind, placebo-controlled fashion, 81 participants completed the study and ingested either a high dose of Phase 2 (1000 mg, WKBE HIGH), a low dose (700 mg, WKBE LOW), or a matching placebo (microcrystalline cellulose, PLA) three times a day, 30 min before meals, for 12 weeks during a calorie restricted diet. In a dose-dependent manner, Phase 2 significantly reduced body weight, fat mass, BMI, waist, hip and in the WKBE HIGH group thigh circumference. Phase 2 is an effective and safe supplement aiding weight and fat loss. ClinicalTrials.gov identifier NCT02930668.

## Introduction

Overweight and obesity are a global public health crisis with 2.5 billion adults being overweight, including 890 million living with obesity in 2022^1^. The worldwide number of people with overweight and obesity is growing dramatically and adolescent obesity has quadrupled since 1990^1^. Reason for the increasing prevalence of people being overweight and/or obese are multifaceted, including reduced energy expenditure (reduced physical activity, sedentary lifestyle), increased energy intake (increased portion sizes, processed foods), environmental factors, genetics, lack of sleep, socioeconomic factors (including access to and ability to afford healthy foods and safe places to be active) and increased life expectancy^2^. Being overweight and obese increases the risk for many health issues, including type 2 diabetes^3^, high blood pressure^4^, heart disease^5^ or osteoarthritis^6^. In addition, people with weight issues have an increased risk of mental health problems including mood disorders^7^.

Carbohydrates are the main source of calories in the diet. Depending on the number of sugar units and how they are chemically bonded, carbohydrates are categorized in sugars, starches, and fibers. Starches need to be broken down into sugars by digestive enzymes before they can be absorbed. During digestion amylase breaks down the polysaccharide starch into oligosaccharides, which are further broken down by glucosidase into monosaccharides. The absorption of starch can be reduced in the gastrointestinal tract by inhibiting the enzymes that break down complex carbohydrates. Glycoproteins, which can be naturally found in the seed of common beans (*Phaseolus* spp.)^8^, prevent the digestion of starch through binding non-covalently via hydrophobic interaction to the active site of alpha-amylase. Of the three alpha-amylase inhibitor isoforms (Alpha-AI1, Alpha-AI2, and Alpha-AIL), Alpha-AI1 shows bioactivity in humans^9,10^. White kidney beans (*Phaseolus vulgaris* L.) are a natural source of Alpha-AI1, and white kidney bean extract (WKBE) has been shown to reduce glucose absorption from foods containing starch^11^.

When given in the diet or as a dietary supplement, WKBEs have beneficial effects on weight management when combined with a healthy diet and exercise by reducing calorie intake, however, potency and the way these extracts are being produced seems to have an impact on their efficacy. A systematic review and meta-analysis of randomized clinical trials of WKBE showed a significant reduction in body fat, however, failed to show significant effects on weight loss^12^. However, a meta-analysis of studies using a specific proprietary aqueous extract from whole dried white kidney beans standardized by its alpha-amylase inhibitor activity (at least 3000 alpha-amylase inhibiting units (AAIU), Phase 2) found statistically significant effects of Phase 2 supplementation on body weight and body fat^13^. Phase 2, also marketed under the brand names Glucosanol, Glycolite, PhaseLite and Starchlite, consists of *Phaseolus vulgaris* extract and Gum Arabic. A proprietary manufacturing process of Phase2 results in an increased stability in acidic conditions of the stomach and duodenum. In vitro, Phase 2 maintains its alpha-amylase inhibitory activity after being exposed to acidic conditions mimicking those of the gastrointestinal tract compared to generic extracts. The present study aims at expanding the clinical evidence of Phase 2 on weight management and safety in subjects with overweight and moderate obesity.

## Methods

### Study design

We conducted a 14 week, double-blind, randomized, placebo-controlled trial. The study was approved (IRB number: EA1/074/16; Date of Approval: 04/18/2016) by the Ethics Committee of the Charité Universitätsmedizin Berlin (Charitéplatz 1, 10117 Berlin, Germany). The intervention period lasted 12 weeks, with five visits for each subject. The screening visit was followed by a 2 week run-in period. The 12 week treatment period comprised the baseline visit (week 0), and visits after 4 and 8 and 12 weeks.

During screening, subjects received instruction on how to maintain a nutritionally balanced, hypocaloric diet providing approximately 45–60% of energy from carbohydrates^14^ and 20–30% of energy from fat^15^. Individual energy requirements were calculated based on BMI and reported activity levels and reduced by 20%. A correction factor was applied to adjust the energy requirement for differences in physical activity. Throughout the study, subjects documented their calorie intake and physical activity on two weekdays and one day of the weekend per week in a diary.

At each visit post-screening, investigators assessed body weight and body composition, blood pressure and pulse rate, adverse events, concomitant treatment and therapies, and subjects filled out the Global Physical Activity Questionnaire (GPAQ). Blood draws were collected following an overnight fast at screening and week 12 and were assessed for the following: total cholesterol, low-density lipoprotein (LDL), high-density lipoprotein (HDL), hemoglobin A1c (HbA1c), hematocrit, erythrocytes, thrombocytes, leukocytes, mean cell volume, mean corpuscular hemoglobin, alanine amino transferase, aspartate amino transferase, creatinine, and urea.

### Participants

The present study aimed to include 90 healthy, male, and female subjects with overweight and moderate obesity. Participants were between 18 and 65 years old, with a BMI of 25 to 34.9 kg/m^2^, healthy, and desiring to lose weight. They were required to be consistent in their daily consumption of three main meals, have maintained a stable body weight prior to study, avoid other weight loss products/programs, follow the diet recommended in the study, keep habitual physical activity level, complete diaries and questionnaires and for women of childbearing potential, to use contraception methods. Exclusion criteria included allergies to the investigational product components or food, significant disorders/surgery within the last six months, eating disorders within the last year, medical implants, regular anticoagulant use, regular medication, or supplementation within the last three months or during the study that could influence body weight or gastrointestinal functions or used for weight management, use of any supplements or natural health products during the study, vegetarian, vegan or macrobiotic diets, recent smoking cessation, pregnancy or nursing, substance abuse, clinically relevant excursions of safety laboratory parameters, inability to comply with study requirements and participation in another clinical study during the study period. Following the 2 week run-in period, the participants had to meet the randomization criteria: no change in body weight or reduction up to maximally 3 kg, readiness and ability to comply with study requirements and relevant inclusion and exclusion criteria met. This clinical study was conducted in accordance with the principles of the World Medical Association (Declaration of Helsinki, version 2013) and the EU recommendations for Good Clinical Practice (CPMP/ICH/135/95), ICH E6(R1). Participation in the study was based upon written informed consent by the participant following written and oral information by the investigator regarding nature, purpose, consequences, and possible risks of the clinical study.

### Anthropometric and body composition measurements

Body weight (kg), body fat content (%), body fat mass (kg) and fat free mass (kg) were assessed by bio-impedance analysis (BIA) method and were measured using validated electronic weighing scales according to the Body composition analyzer BC-420MA instruction manual (Tanita Corporation, 2005). Waist circumference (cm) was measured at the level midway between the lateral lower rib margin and the iliac crest (WHO, 1995). Hip circumference (cm) was measured as the maximal circumference over the buttocks (WHO, 1995). Thigh circumference (cm) was measured as the maximum circumference of the thigh.

### Dosing and blinding

During the 12 week intervention, subjects were instructed to orally consume a total of 6 capsules daily: two capsules 30 min before each of the three main meals (breakfast, lunch, and dinner) with a glass of water (150 mL). Each capsule contained 350 mg Phase 2 (low dose, WKBE LOW) or 500 mg Phase 2 (high dose, WKBE HIGH) or microcrystalline cellulose (PLA). All capsules were identical in appearance, texture, taste, and smell, as well as packaging and labelling, so that study participants, investigators and study personnel were blinded to treatment assignment.

### Compliance

Overall compliance was defined as the total number of capsules taken divided by the total number of capsules to be taken, multiplied by 100. The difference between the total number of capsules dispensed and the total number of capsules returned was used to calculate the total number of capsules used. A subject was classified as non-compliant when consuming less than 80% or more than 120% of the correct quantity of investigational product.

Study duration and calorie intake were also evaluated to assess compliance. With respect to calorie intake, a subject was classified as non-compliant when there was a deviation of more than 20% of the indicated individual diet plan within the overall intervention period based on subject diary entries. Throughout the entire study period (including run-in phase), subjects were to record their daily intake of calorie-containing food and beverages (24 h) for 2 weekdays and one weekend day each week (randomly selected) in a subject diary.

### Statistical analysis

The sample size calculation for this study was based on data from a previous study that used the same product^16^. Phase 2 consumption over three months resulted in a 3.5% reduction in body weight^17^, which is considered physiologically relevant. According to estimates from the Diabetes Prevention Program lifestyle intervention data, a reduction of one kilogram in body weight is associated with a 16% reduction in diabetes risk, when at least 2% of body weight loss from baseline is achieved^17^. The sample size calculation for the current study was performed for the difference in body weight changes after 12 weeks compared to baseline, considering two defined groups: 500 mg Phase 2 versus placebo (primary outcome). The calculation, using two-sided testing with a power of 90% and a significance level of 0.05, resulted in 28 subjects per group. Accounting for a 20% dropout rate, the total number per arm was set to be 36. In the lower dosage group, 18 subjects were randomized, with the randomization ratio between the three study arms fixed at 2:1:2: 1) 500 mg Phase 2 (WKBE HIGH), 2) 350 mg Phase 2 (WKBE LOW), and 3) Placebo (PLA). Thus, a total of 90 subjects were randomized for the study. A central stratified block randomization was used, stratified according to center, gender and BMI (< 30 and > 30) at baseline visit (V2), when it was determined that the inclusion and randomization criteria had been fulfilled and the exclusion criteria not violated. The random allocation sequence was generated by the responsible personal (acromion GmbH, Frechen, Germany), not involved in the clinical trial, by the PLAN procedure using the SAS software package version 9.4 under the Microsoft Windows 7 Professional operating system. The randomization list was provided to the independent pharmacist (Stier Pharmacy, Berlin, Germany) that carried out the study material labeling and provided the labeled blinded study materials.

The change in body weight, both absolute (kg) and relative (%), BMI, body fat mass, waist, hip, and thigh circumferences between 0 and 12 weeks were analyzed using the univariate exact Wilcoxon-Mann–Whitney test with a 5% significance level. A two-way mixed repeated measures ANOVA with baseline as covariate was also conducted to follow the EMEA guideline CPMP/EWP/2863/99. Additional secondary outcomes were to examine the change in weight loss at 4 and 8 weeks from baseline for in addition to the proportion of subjects who lost at least 3% and 5% of baseline body weight after 4, 8 and 12 weeks between the three groups, compared to baseline. Statistical tests for secondary outcomes were exploratory without adjustment for multiple comparisons. As the primary outcome and some secondary outcomes were measured at consecutive time points, statistical methods accounting for time-dependency were applied. This included a multivariate nonparametric analysis of longitudinal data in a two-factorial design and a nonparametric analysis (i.e., ANOVA) of covariance for repeated measurements with baseline as covariate in cases where baseline values impacted subsequent measures. Multivariate generalized estimating equations (GEE) were used to model the relationship between outcome parameters and potential risk factors in a generalized regression model over time. Multivariate longitudinal analysis was analyzed via R (Version 3.4.0) and SAS (Version 9.4), multivariate covariance analyses for longitudinal data via SAS and univariate nonparametric analyses and GEE via SPSS (Versions 22 and 24). Data are presented as means ± SDs.

## Results

**Figure 1:**
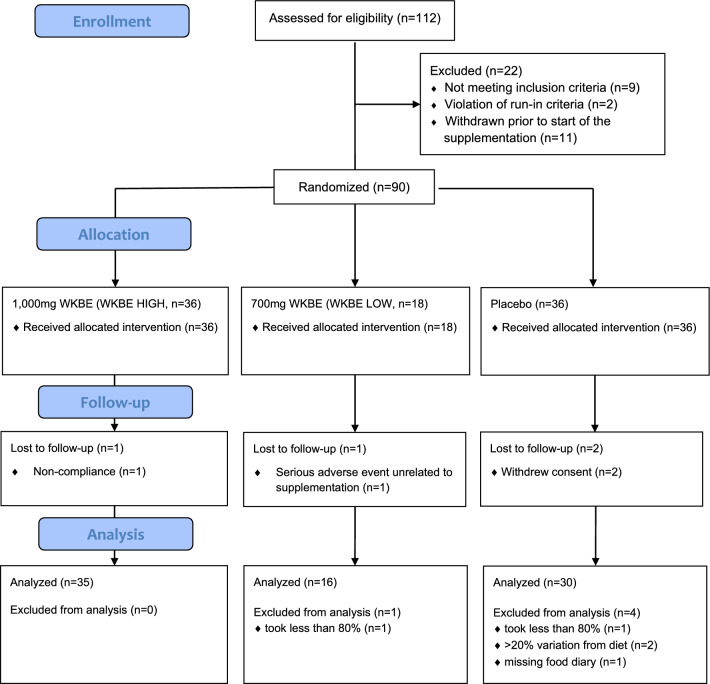
CONSORT Flow Diagram.

112 subjects were screened from May 2016 to July 2016 at two centers in Berlin, Germany. The first subject was enrolled in May 2016 and the last subject completed the study in November 2016. Nine subjects were not randomized due to exclusion criteria (screening failures), and 2 were not randomized due to the violation of the randomization criteria (run-in failures). 11 subjects had withdrawn from the study before supplementation was started. 90 subjects received treatment, with 36 receiving a high dose of Phase 2 (1000 mg, WKBE HIGH), 18 receiving a low dose (700 mg, WKBE LOW), and 36 receiving a matching placebo (microcrystalline cellulose, PLA) three times a day for 12 weeks. A total daily dose of 3000 mg, taken in three separate doses of 1000 mg with the three major meals in the commonly used dose in WKBE studies^13^. Alternate dosing from previous studies includes 1500 mg twice per day per day, and one study used a lower daily dose of 2000 mg, as twice 1000 mg per day at breakfast and lunch^13^. The 2000 mg dose study showed only minimal benefits, which could be due to the low dose, or short duration of the study (4 weeks). We used the validated 3000 mg as the high dose and 2100 mg as the low dose, as 2000 mg was the previously studied lowest dose. Their baseline characteristics can be found in Table 1. Of these, four subjects terminated the study prematurely. One subject assigned to verum high dose withdrew due to non-compliance, while two subjects assigned to placebo withdrew due to withdrawal of consent. Another subject assigned to verum low dose withdrew due to a serious adverse event unrelated to supplement intake. All remaining 86 subjects were included in the full analysis set (FAS). Further, five more subjects were excluded from final analysis. Two subjects, one each from WKBE LOW and PLA, took less than 80% of the correct supplementation quantity. Two subjects from the PLA group deviated from the prescribed diet plan by more than 20% in caloric intake. Another subject from the PLA group had no available data on caloric intake (missing diary). Thus, 81 subjects were included in the valid case analysis set (VCAS) **(**Fig. 1**)**.

**Table 1:** Baseline participant characteristics. Means ± SDs.

	WKBE HIGH (n = 36)	WKBE LOW (n = 18)	PLA (n = 36)
Male/female (n)	12/24	6/12	12/24
Age (years)	48 ± 12	51 ± 12	46 ± 11
Body weight (kg)	85.9 ± 12.5	86.3 ± 11.4	85.3 ± 11.0
BMI (kg/m^2^)	29.5 ± 2.3	30.1 ± 2.8	30.0 ± 2.3
Waist circumference (cm)	101.1 ± 8.6	103.8 ± 8.6	103.0 ± 10.4
Hip circumference (cm)	105.2 ± 7.9	105.7 ± 9.2	107.8 ± 8.4
Thigh circumference (cm)	62.8 ± 5.0	63.0 ± 6.5	62.8 ± 5.5

### Changes in body weight, BMI, and body composition

#### Changes in body weight at 4, 8 and 12 weeks

**Figure 2:**
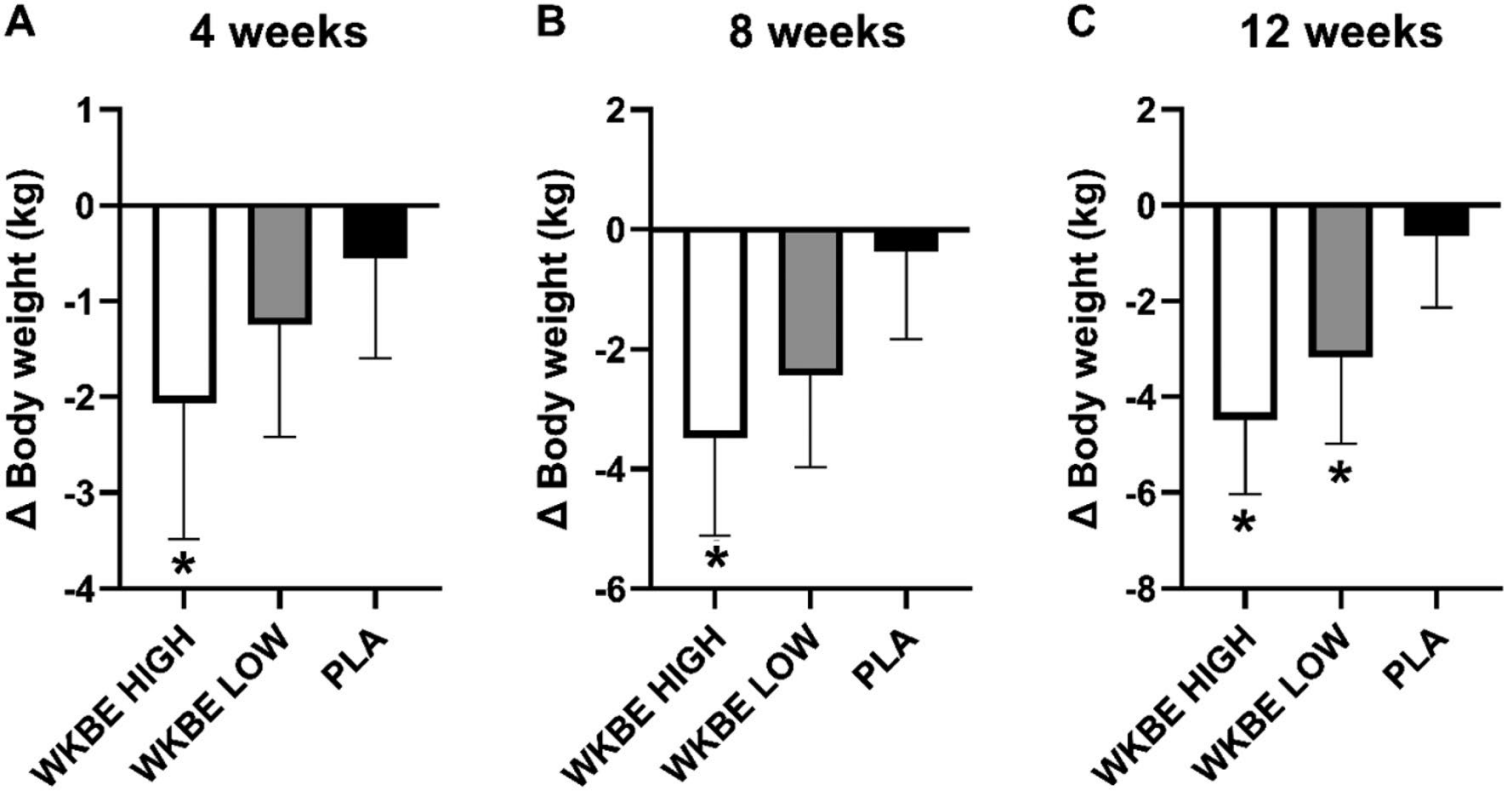
Changes in body weight at (**A**) 4 weeks (**B**) 8 weeks and (**C**) 12 weeks in response to supplementation with white kidney bean extract. *WKBE significantly different than PLA (p < 0.001). Means ± SD.

At 4 weeks **(**Fig. 2A**)**, body weight was significantly reduced in WKBE HIGH (2.06 ± 1.42 kg), when compared to PLA (0.55 ± 1.05 kg; P < 0.001). At 8 weeks **(**Fig. 2B**)**, body weight was significantly reduced in WKBE HIGH (3.48 ± 1.63 kg), when compared to PLA (0.36 ± 1.48 kg; P < 0.001)*.* The change in body weight at 12 weeks **(**Fig. 2C**)** was significantly reduced in both (P < 0.001) WKBE HIGH (4.48 ± 1.56 kg) and WKBE LOW (3.18 ± 1.74 kg), when compared to PLA (0.54 ± 1.46 kg) that was maintained when considering baseline values as a covariate (P < 0.001). WKBE HIGH induced greater body weight loss compared to WKBE LOW that was maintained when adjusting for baseline values (P = 0.001).

#### Changes in BMI 4, 8 and 12 weeks

**Figure 3:**
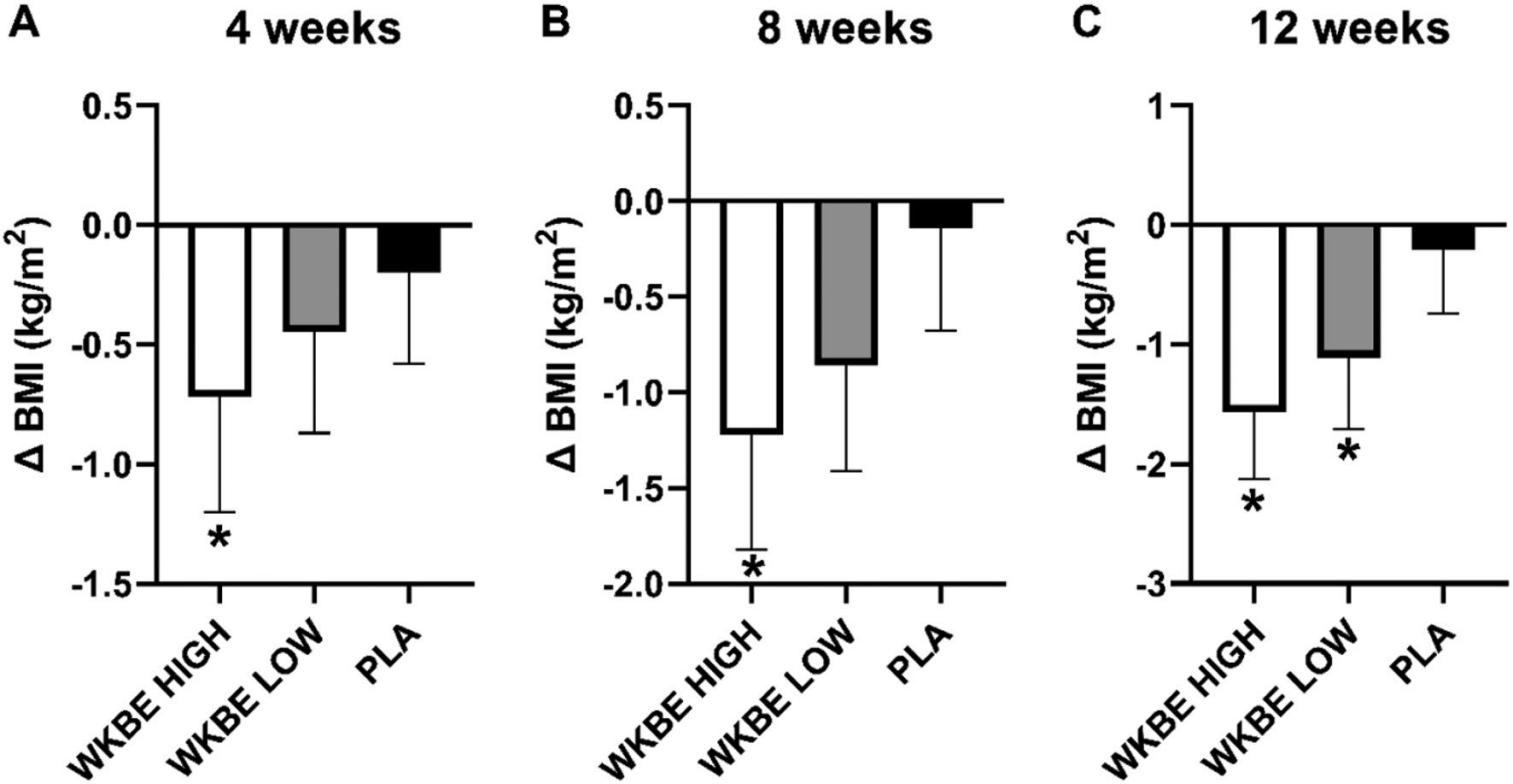
Changes in BMI at (**A**) 4 weeks (**B**) 8 weeks and (**C**) 12 weeks in response to supplementation with white kidney bean extract. *WKBE significantly different than PLA (p < 0.001). Means ± SD.

At 4 weeks **(**Fig. 3A**),** WKBE HIGH had a statistically significant reduction in BMI compared to PLA (0.72 ± 0.48 kg/m^2^ vs. 0.20 ± 0.38 kg/m^2^; P < 0.001). At 8 weeks **(**Fig. 3C**)**, WKBE HIGH also had a statistically significant reduction in BMI compared to PLA (1.22 ± 0.60 kg/m^2^ vs. 0.14 ± 0.54 kg/m^2^; P < 0.001). WKBE HIGH further showed a statistically significant reduction in BMI **(**Fig. 3C**)** compared to PLA after 12 weeks (1.56 ± 0.57 kg/m^2^ vs. 0.21 ± 0.53 kg/m^2^; P < 0.001). Similarly, WKBE LOW demonstrated a statistically significant reduction in BMI compared to the PLA group after 12 weeks (1.11 ± 0.60 kg/m^2^; P < 0.001). WKBE HIGH reduced BMI to a greater extent than WKBE LOW (P = 0.004).

### Body composition

#### Fat mass at 4, 8 and 12 weeks

**Figure 4:**
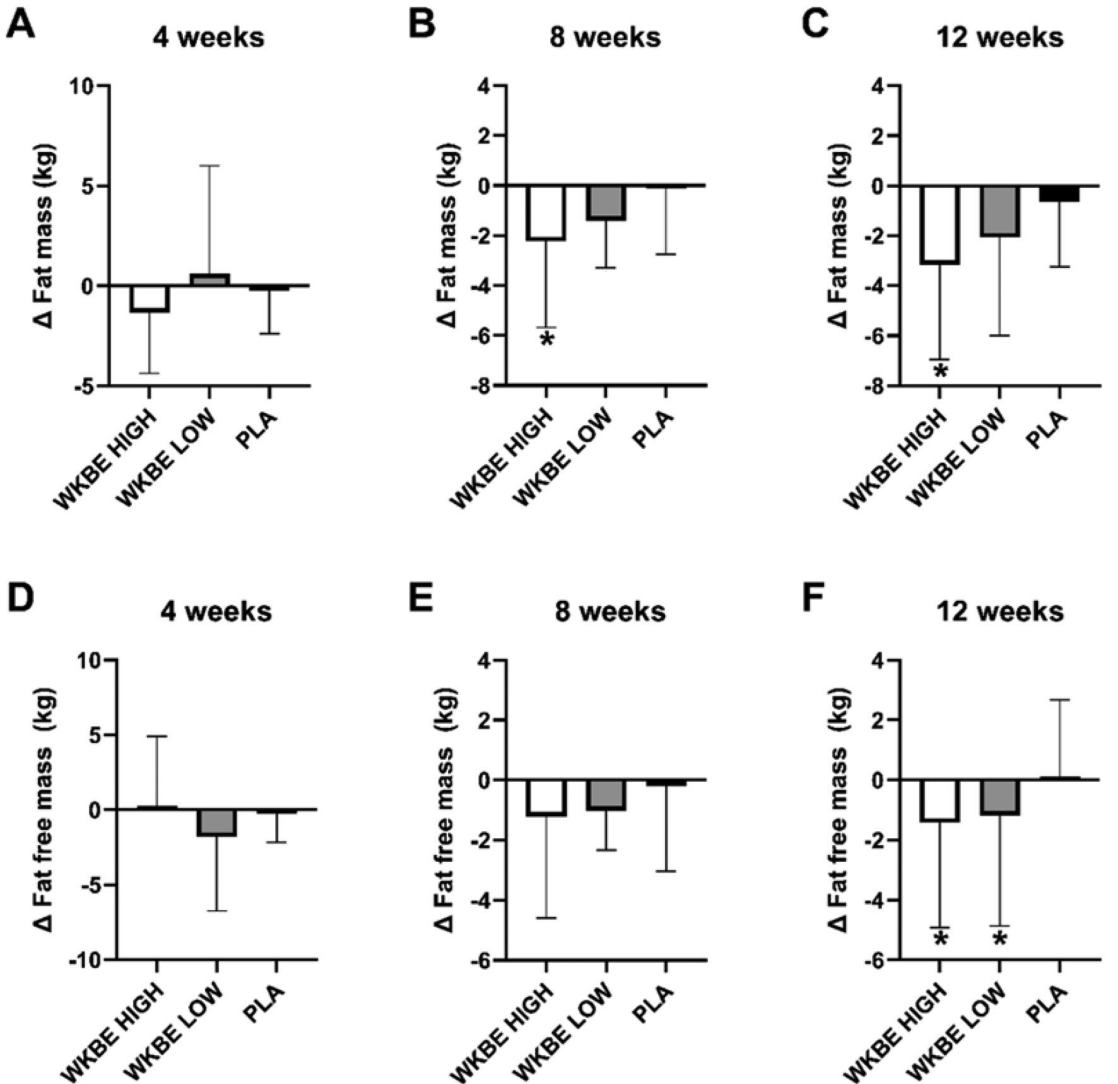
Changes in fat mass at (**A**) 4 weeks (**B**) 8 weeks and (**C**) 12 weeks and fat free mass at (**D**) 4 weeks (**E**) 8 weeks and (**F**) 12 weeks in response to supplementation with white kidney bean extract. *WKBE groups significantly different than PLA (p < 0.05). Means ± SD.

At 4 weeks **(**Fig. 4A**)**, there was no statistically significant difference in fat mass between WKBE HIGH and PLA groups (P > 0.14) and from baseline (P > 0.29). However, at 8 weeks **(**Fig. 4B**)**, fat mass was significantly reduced in WKBE HIGH compared to PLA (2.22 ± 3.46 kg vs 0.11 ± 2.65 kg; P = 0.002).

There was a significant difference in changes in body fat mass **(**Fig. 4C**)** between WKBE HIGH and PLA after 12 weeks compared to baseline. WKBE HIGH reduced fat mass to a greater extent than PLA (3.17 ± 3.78 kg vs 0.65 ± 2.60 kg; P = 0.002). After 12 weeks, WKBE LOW reduced body fat mass by 2.06 ± 3.92 kg but was not significantly different from PLA (P = 0.268).

#### Fat free mass at 4, 8 and 12 weeks

At 4 and 8 weeks **(**Fig. 4D-E**)**, fat free mass changes from baseline were not significantly different between WKBE and PLA groups (P = 0.694 and P = 0.098 respectively). However, after 12 weeks, fat free mass **(**Fig. 4F**)** was significantly reduced by 1.42 ± 3.49 kg in the WKBE HIGH group compared to an increase of 0.12 ± 2.56 kg in PLA (P = 0.027). Similarly, significant reduction was observed in the WKBE LOW versus PLA after 12 weeks (1.17 ± 3.69 kg; P = 0.020).

#### Changes in body fat (%)

After 4 and 8 weeks, the difference in body fat content between the 3 groups was not significant (P = 0.354 and P = 0.067; respectively). After 12 weeks, the reduction in body fat content was greater in the WKBE HIGH (1.91 ± 4.45%) compared to PLA (0.60 ± 3.06%), but this difference was not statistically significant (P = 0.065). For WKBE LOW, the reduction in body fat content was 2.43 ± 5.59% after 12 weeks of supplementation, which was not significantly different from PLA (P = 0.395).

## Responders

### Responders who lost at least 3% and 5% of body weight at weeks 4, 8 and 12

**Figure 5:**
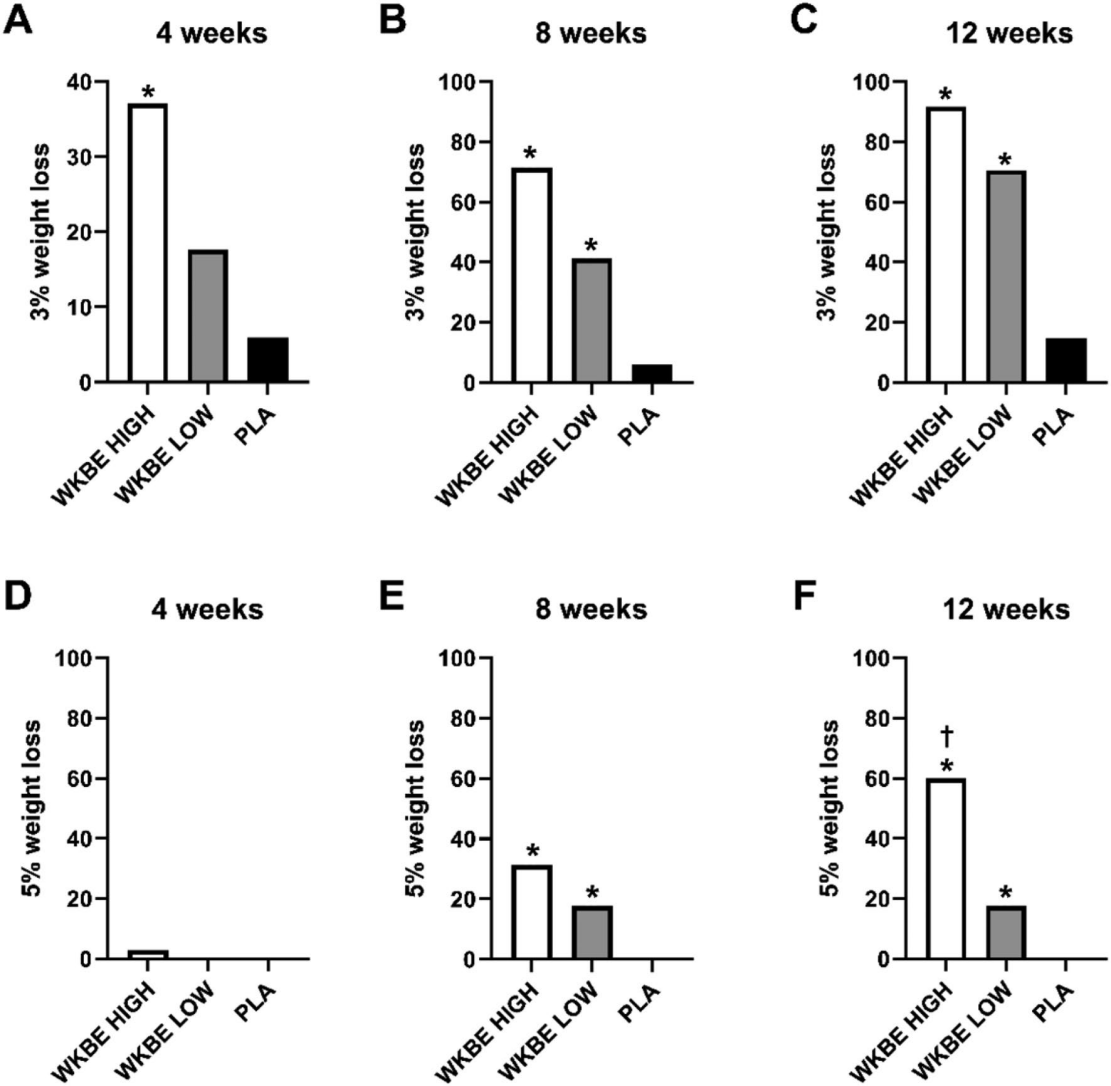
The proportion of participants who lost at least 3% of body weight at (**A**) 4 weeks (**B**) 8 weeks and (**C**) 12 weeks and 5% of body weight at (**D**) 4 weeks (**E**) 8 weeks and (**F**) 12 weeks in response to supplementation with white kidney bean extract. †WKBE HIGH significantly different than WKBE LOW. *WKBE groups significantly different than PLA (p < 0.05)..

At 4 weeks **(**Fig. 5A**)**, there was a significant difference (P = 0.003) between the proportion of participants who lost at least 3% of body weight in WKBE HIGH (37.1%) but not WKBE LOW (17.6%; P = 0.32) when compared to PLA (5.9%), with no significant differences between WKBE HIGH and WKBE LOW (P = 0.21). At 8 weeks **(**Fig. 5B**)**, there was a significant difference between the proportion of participants who lost at least 3% of body weight in both WKBE HIGH (71.4%) and WKBE LOW (41.2%) when compared to PLA (5.9%) (P < 0.001 and P = 0.004, respectively), while no significant differences between WKBE HIGH and WKBE LOW (P = 0.067). At 12 weeks, there was a significant difference between the proportion of participants who lost at least 3% **(**Fig. 5C**)** of body weight in both WKBE HIGH (91.7%) and WKBE LOW (70.6%) when compared to PLA (14.7%) (P < 0.001 in both cases), with no significant differences between WKBE HIGH and WKBE LOW (P = 0.096).

There was no statistically significant difference between the 3 study groups in proportion of subjects with at least 5% body weight loss at 4 weeks **(**Fig. 5D**)**. At 8 weeks **(**Fig. 5E**)**, there was a significant difference between the proportion of participants who lost at least 5% of body weight in both WKBE HIGH (31.2%) and WKBE LOW (17.6%) when compared to PLA (0%) (P < 0.001 and P = 0.033 respectively), with no significant differences between WKBE HIGH and WKBE LOW (P = 0.34). At 12 weeks, there was a significant difference between the proportion of participants who lost at least 5% **(**Fig. 5F**)** of body weight in both WKBE HIGH (60.0%) and WKBE LOW (17.6%) when compared to PLA (0%), with P < 0.001 and P = 0.033 respectively, the difference was also significant between WKBE HIGH and WKBE LOW (P = 0.007).

### Body circumferences

#### Waist circumference at weeks 4, 8 and 12

**Figure 6:**
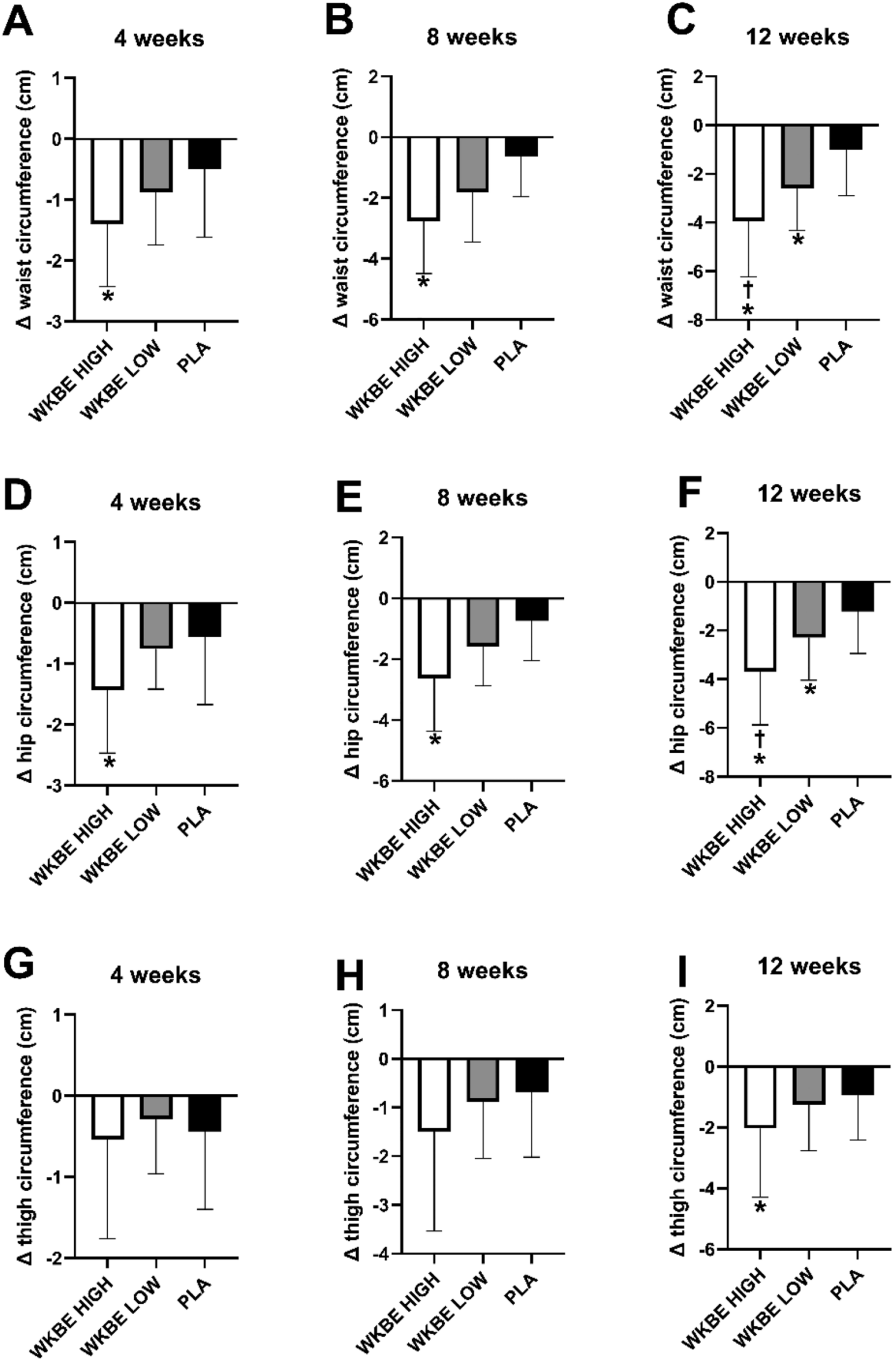
Changes in waist circumference at (**A**) 4 weeks (**B**) 8 weeks and (**C**) 12 weeks, hip circumference at (**D**) 4 weeks (**E**) 8 weeks and (**F**) 12 weeks and thigh circumference at (**G**) 4 weeks (**H**) 8 weeks and (**I**) 12 weeks in response to supplementation of white kidney bean extract. †WKBE HIGH different than WKBE LOW. *WKBE groups significantly different than PLA (p < 0.05). Means ± SD.

At 4 weeks **(**Fig. 6A**)**, WKBE HIGH significantly reduced waist circumference by 1.40 ± 1.03 cm compared to the 0.50 ± 1.11 cm reduction seen in PLA (P < 0.001). The difference in waist circumference changes between the three groups was statistically significant (P < 0.001). After 8 weeks **(**Fig. 6B**)**, WKBE HIGH significantly reduced their waist circumference by 2.77 ± 1.72 cm compared to the 0.62 ± 1.33 cm reduction seen in the PLA group (P < 0.001). The difference in waist circumference changes between the three groups was statistically significant (P < 0.001). After 12 weeks, WKBE HIGH reduced waist circumference **(**Fig. 6C**)** by 3.94 ± 2.29 cm compared to 2.59 ± 1.73 cm (P = 0.03) and 1.00 ± 1.89 cm (P < 0.001) reduction seen in WKBE LOW and PLA group, respectively. WKBE LOW reduced waist circumference more than the PLA group (P = 0.001).

#### Hip circumference at 4, 8 and 12 weeks

At 4 weeks **(**Fig. 6D**)**, hip circumference was significantly reduced by 1.43 ± 1.04 cm in the WKBE HIGH compared to 0.56 ± 1.11 cm in PLA (P < 0.001). The difference in hip circumference changes between the three groups was statistically significant (P < 0.001). At 8 weeks **(**Fig. 6E**)**, WKBE HIGH significantly reduced their hip circumference by 2.63 ± 1.73 cm compared to the 0.74 ± 1.31 cm reduction seen in the PLA group (P < 0.001). The difference in hip circumference changes between the three groups was statistically significant (P < 0.001). After 12 weeks, WKBE HIGH group significantly reduced hip circumference **(**Fig. 6F**)** by 3.69 ± 2.19 cm compared to 2.29 ± 1.76 cm (P = 0.022) and 1.21 ± 1.74 cm (P < 0.001) reduction seen in WKBE LOW and PLA, respectively. WKBE LOW reduced hip circumference more than the PLA group (P = 0.021).

#### Thigh circumference at 4, 8 and 12 weeks

The differences in thigh circumference changes from baseline were not significant at weeks 4 and 8 **(**Fig. 6G-H**)** between groups (P = 0.84 and P = 0.06 respectively). After 12 weeks, thigh circumference **(**Fig. 6I**)** significantly decreased by 2.03 ± 2.27 cm in WKBE HIGH, compared to 0.94 ± 1.48 cm in PLA (P = 0.028). In WKBE LOW, thigh circumference was reduced by 1.24 ± 1.52 cm, but was not different from PLA (P = 0.406). There was no significant difference between the WKBE HIGH and WKBE LOW (P = 0.285).

#### Waist-to-hip ratio

Waist-to-hip ratio changes from baseline were not significantly different between all the study groups at week 4, 8 and 12 (P ≥ 0.21; data not shown).

### Diet and exercise

#### Calorie intake and physical activity

At every week during the 12 weeks intervention period, there was no statistically significant difference between the WKBE HIGH, WKBE LOW and PLA groups in changes in daily calorie intake (P > 0.05). Physical activity assessed per GPAQ was similar between groups at weeks 4, 8 and 12 (P ≥ 0.26) during the study.

### Clinical laboratory evaluation

Changes in total cholesterol, LDL, HDL and HbA1c were not different between WKBE HIGH and PLA (P ≥ 0.07; data not shown) or WKBE LOW and PLA (P ≥ 0.36; data not shown) at week 12 compared to baseline. Changes in hemoglobin, hematocrit, erythrocytes, thrombocytes, leukocytes, mean cell volume, mean corpuscular hemoglobin, alanine amino transferase, aspartate amino transferase, alkaline phosphatase, gamma glutamyl transferase, creatinine and urea were not different between groups (P ≥ 0.15; data not shown) at week 12 compared to baseline.

### Adverse events (AE)

During the study 19 AE in 19 subjects (19 of 90; 21.1%) were documented, including 1 serious AE: 6 subjects in the WKBE HIGH group (6 of 36; 16.7%: distortion trauma ankle joint left side (1), bacterial bronchitis (1), bruise knee joint right side (1), respiratory infection (1), angina of the salpingopalatine fold (1), urinary tract infection (1)), 5 subjects in the WKBE LOW group (5 of 18; 27.8%: meteorism (1), pharyngitis (2), urinary tract infection (1), pulmonary embolism (1)), and 8 subjects in the PLA group (8 of 36; 22.2%: upper respiratory tract infection (2), otitis externa left side (1), acute gastritis (1), urinary tract infection (1), acute bronchitis (1), distortion knee joint right side (1), pharyngitis(1)). There were no differences between study groups in the percentages of subjects with AE (P = 0.631). 17 AEs were of light, 1 of moderate intensity. In one case the AE “meteorism” was assessed as “possibly related”, for all other AEs, including the serious AE, the causal relationship was “unlikely related” to the intake of the study material.

## Discussion

We conducted a 12-week, double-blind, placebo-controlled randomized trial to investigate the effects of WKBE supplementation (Phase 2) on body weight, BMI and body composition. Our findings demonstrate significant reductions in body weight, BMI, and fat mass in the WKBE HIGH (Phase 2 3 g/day) group compared to the placebo group with the divergence between WKBE HIGH and LOW (Phase 2 2.1 g/day) occurring at 12 weeks. The result of our study confirmed the outcome of a previous 12-week study with same product using a hypocaloric (500 kcal less than their basal energy needs per day)^16^. WKBE LOW also exhibited significant reductions in these outcomes, albeit to a lesser extent than WKBE HIGH. Furthermore, WKBE supplementation resulted in a higher proportion of responders who achieved at least 3% and 5% body weight loss compared to the placebo group. We observed significant reductions in waist and hip circumferences in the WKBE HIGH group compared to both WKBE LOW and placebo groups at 12 weeks. Calorie intake and physical activity levels did not differ significantly between the WKBE and placebo groups during the intervention period.

In the safety assessment of the present study, we did not identify any issues. All participants as well as investigators rated the tolerability of WKBE and placebo as good or very good in the global assessment of tolerability. Only one case (meteorism) of adverse event was assessed as possible related. Additionally, there were no significant differences between the groups in various clinical laboratory parameters. These findings suggest that WKBE supplementation may effectively contribute to weight loss without affecting clinical laboratory measures.

The mechanisms underlying the observed weight loss effects of both high and low doses of WKBE in overweight adults is based on the inhibition of alpha-amylase, an enzyme responsible for the breakdown of complex carbohydrates into simpler sugars^18^. WKBE contains a natural compound called phaseolamin, which has been shown to inhibit alpha-amylase activity^19^, leading to reduced carbohydrate digestion and absorption^20^. This inhibition may result in decreased postprandial glucose and insulin levels^21^, potentially reducing de novo lipogenesis, and facilitating weight loss^22^. Furthermore, WKBE has been suggested to modulate gut microbiota composition in preclinical^23^ and clinical^24^ models, which can influence energy metabolism^25^ and adiposity^26^. It is possible that WKBE supplementation may promote the growth of beneficial bacteria, leading to enhanced energy expenditure and improved metabolic homeostasis^27^. Gum arabic in Phase 2 is used as a technical aid, for its emulsifying, stabilizing, binding, and shelf-life enhancing properties, not as an additive or synergistic active ingredient. While high intake of dietary fiber, including 30 g of gum Arabic^28^, is associated with anti-obesity effects, the amounts of gum arabic from the intake of 3 or 2.1 g of Phase 2 per day is too low to have a beneficial effect.

The identification of responders who achieved at least 3% and 5% body weight loss in our study may hold significant clinical implications^29^. At 12 weeks, a significantly higher proportion of participants in both the WKBE HIGH and WKBE LOW groups achieved these weight loss milestones compared to the placebo group. Modest weight loss of 3% has been shown to lead to improvements in metabolic parameters, such as blood pressure, blood glucose, and lipid profiles^30^. WKBE HIGH achieved 3% weight loss within 4 weeks. Furthermore, achieving a 5% weight loss has been linked to a reduced risk of developing chronic diseases, especially in individuals predisposed to type 2 diabetes and cardiovascular diseases^31^. Thus, the increased proportion of responders achieving these weight loss thresholds, especially with WKBE HIGH at 12 weeks, suggests that WKBE supplementation may offer a promising approach to aid in weight management and improve overall health outcomes in overweight individuals. However, further research is needed to explore the long-term effects and sustainability of these weight loss outcomes. A limitation of our study was the limited subject number in the WKBE LOW group. Strengths included multimodal assessment of body composition, mixed gender, and multiple longitudinal measurements allowing to assess the minimum time needed to see beneficial changes in weight management.

The reductions in waist circumferences observed in our study hold clinical significance particularly when considered alongside the body weight and BMI data. After 12 weeks the WKBE HIGH group observed reductions in waist, hip, and thigh circumferences compared to WKBE LOW and the placebo group. Reductions in body circumferences are indicative of a decrease in adipose tissue deposition and localized fat accumulation^32^. Waist circumference, in particular, is a strong indicator of people with abdominal obesity and is closely associated with an increased risk of metabolic disorders, including cardiovascular diseases^33^, type 2 diabetes^34^, and hypertension^35^. Importantly, the WKBE HIGH demonstrated reductions in waist and hip circumference at 4 weeks which was further reduced by week 12. The observed loss of fat free mass in the WKBE HIGH and WKBE LOW groups at 12 weeks, but not at 4 and 8 weeks, is likely attributable to the participants' consumption of a low-calorie diet over time^36^. It is well established that the preservation of lean body mass is crucial for maintaining metabolic health and physical functionality^37^. Collectively, the reductions in body circumferences observed alongside the body weight and BMI data suggest that WKBE supplementation may contribute to favorable changes in body composition and the reduction of health risks in people with obesity.

## Conclusion

In conclusion, our findings from this 12 week double-blind placebo-controlled randomized trial demonstrate the potential efficacy of WKBE (Phase 2) supplementation in promoting weight loss, BMI, and reducing body circumferences in adults with overweight and moderate obesity. Both the WKBE HIGH and WKBE LOW groups exhibited significant reductions in body weight, BMI, and fat mass compared to the placebo group. Furthermore, WKBE supplementation resulted in a higher proportion of participants achieving clinically significant weight loss thresholds of 3% and 5% of body weight. Our findings contribute to the growing body of evidence highlighting the use of WKBE supplementation as an adjunctive approach for weight management.

## Data Availability

Datasets can be accessed from the corresponding author upon request.
